# Tissue Metabolic Responses to Salt Stress in Wild and Cultivated Barley

**DOI:** 10.1371/journal.pone.0055431

**Published:** 2013-01-31

**Authors:** Dezhi Wu, Shengguan Cai, Mingxian Chen, Lingzhen Ye, Zhonghua Chen, Haitao Zhang, Fei Dai, Feibo Wu, Guoping Zhang

**Affiliations:** 1 Department of Agronomy, Key Laboratory of Crop Germplasm Resource of Zhejiang Province, Zhejiang University, Hangzhou, China; 2 School of Science and Health, University of Western Sydney, Penrith, New South Wales, Australia; China Agricultural University, China

## Abstract

A thorough understanding of the mechanisms underlying barley salt tolerance and exploitation of elite genetic resource are essential for utilizing wild barley germplasm in developing barley varieties with salt tolerance. In order to reveal the physiological and molecular difference in salt tolerance between Tibetan wild barley (*Hordeum spontaneum*) and cultivated barley (*Hordeum vulgare*), profiles of 82 key metabolites were studies in wild and cultivated barley in response to salinity. According to shoot dry biomass under salt stress, XZ16 is a fast growing and salt tolerant wild barley. The results of metabolite profiling analysis suggested osmotic adjustment was a basic mechanism, and polyols played important roles in developing salt tolerance only in roots, and high level of sugars and energy in roots and active photosynthesis in leaves were important for barley to develop salt tolerance. The metabolites involved in tolerance enhancement differed between roots and shoots, and also between genotypes. Tibetan wild barley, XZ16 had higher chlorophyll content and higher contents of compatible solutes than CM72, while the cultivated barley, CM72 probably enhanced its salt tolerance mainly through increasing glycolysis and energy consumption, when the plants were exposed to high salinity. The current research extends our understanding of the mechanisms involved in barley salt tolerance and provides possible utilization of Tibetan wild barley in developing barley cultivars with salt tolerance.

## Introduction

Currently, nearly 20% of arable land and 50% of irrigated land in the world are salt-affected, which causes a great threat to agricultural production [1], [2]. The development of salt tolerant varieties is one of the most effective ways for effective utilization of salted soil. However, the progress in developing salt-tolerant crops is significantly hampered by the physiological and genetic complexity of this trait. Hence, understanding of salt-tolerant mechanisms is imperative for crop improvement in salt tolerance.

Barley (*Hordeum vulgare* L.) is a most salt tolerant crop in the grass family [3], and is widely used in physiological and genetic studies of salt tolerance. Especially, wild barley has adapted to a broad range of environments and formed rich genetic diversities for salt tolerance [4]. For instance, Tibetan wild barley (*Hordeum Spontaneum* L.), considered as one of the ancestors of cultivated barley [5], [6], is characterized by wide variation of abiotic tolerance, and some accessions with high tolerance to drought [7], aluminum toxicity [8] and salinity [9] have been identified. In our previous study, we assessed the salt tolerance of around 200 accessions of Tibetan wild barley and identified some elite salt-tolerant accessions (e.g. XZ16 and XZ26) [9], [10].

It is well documented that osmotic stress, ion toxicity and secondary stress (i.e. oxidation), are the three major damages to plants under salt stress [3], [11], [12]. High salt concentration in soils inhibits uptake of water and nutrients by plant roots due to osmotic stress [13]. Direct and excessive entrance of sodium ions into plant cells will cause ion toxicity and imbalance, restraining plant photosynthesis and metabolism [13], [14]. Moreover, large amount of reactive oxygen species (ROS) will be produced in plant cells upon sustaining salt stress, leading to serious damage to plants [11], [15].

On the other hand, plants have developed some mechanisms for salt stress adaptation or tolerance, including tissue tolerance to osmotic stress, ion homeostasis and detoxification [3]. Plants can adjust osmotic stress by accumulating high concentrations of compatible solutes in cytoplasm, There are four main classes of compatible solute in plant cells, including: N-containing compounds (i.e. proline and glycine betaine), sugars (i.e. sucrose and raffinose), straight-chain polyhydric polyols such as mannitol and sorbitol, and cyclic polyhydric alcohols [13], [16], [17]. Many genes that regulate osmolyte synthesis have been identified and their functions were characterized [3], [16], [17]. Δ1-Pyrroline-5-carboxylase synthase (*P5CS*), which regulates proline accumulation, was rapidly induced by salt stress, resulting in enhanced osmotic stress tolerance in *Arabidopsis* [18]. Hong et al. also showed that proline level increased when transgenic tobacco plants carrying a modified *P5CS* gene were exposed to salt stress [19]. Over-expression of choline oxidase (*CodA*) gene increased glycine betaine synthesis, and enhanced tolerance to salt and cold stress in rice [20]. Similarly, exogenous expression of *mt1D*, a gene for mannitol-1-phosphate dehydrogenase, regulated mannitol synthesis, thus enhancing tolerance to water and salinity stresses [21]. For ion homeostasis, sodium efflux and the ability to maintain a high K^+^ concentration were the main strategies for plants to fight against salt stress [11], [14]. Different transporters (*SOS*, *HKT* and *NHX* genes) in plants play fundamental roles in sodium efflux and compartmentation [14], [22], [23]. Further, suffering from long-term salt stress, plants would generate massive reactive oxygen species (ROS), such as O_2_, H_2_O_2_, HO•, O_2_•^−^, which caused secondary stress to plants [15]. Correspondingly, plants have developed the defense system of scavenging ROS, mainly through antioxidative enzymes, such as superoxide dismutase (SOD), ascorbate peroxidase (APX), catelase (CAT), glutathione peroxidase (GPX) [11], [15].

The high-throughput omics analysis, including transcriptomics, proteomics, and metabolomics, will improve comprehensive understanding of salt stress-induced changes in gene-protein-metabolite [24]. Transcriptomics and proteomics analysis have been widely used in salt tolerance studies [24]–[26]. Currently, metabolomics are developed and applied in understanding multiple physiological processes in plants, in combination with other platforms such as transcript profiling and proteomics. Major approaches currently used in plant metabolomics are metabolic fingerprinting, metabolite profiling and targeted analysis, and main analysis methods include gas chromatography-mass spectrometry (GC-MS), liquid chromatography-mass spectrometry (LC-MS), capillary electrophoresis-mass spectrometry (CE-MS), fourier transform-ion cyclotron resonance-mass spectrometry (FT-ICR-MS) and nuclear magnetic resonance (NMR) [27]–[29]. In recent years, metabolomics analysis is being widely used to investigate abiotic stress tolerance of plants [29]–[32]. In *Arabidopsis thaliana*, the metabolite profiling was analyzed in response to temperature [33], salt stress [34], K nutrition [35] and combined stress of high temperature and drought [36]. Metabolome changes were also reported in cultivated barleys in response to boron toxicity [37] and salt stress [38]. Up to now, there is no relevant metobolomics research on wild barley although it harbors numerous elite genetic resources for improvement of abiotic stress tolerance in cultivated barley.

In this study, two wild and two cultivated barley genotypes varying in salinity tolerance were selected and the two salt-tolerant genotypes, a cultivated barley and a Tibetan wild barley, were further used to compare metabolic changes in response to salt stress in tissues using GC-MS. The primary objective of this work is to determine the possible difference of metabolic profiles in response to salt stress between cultivated and wild barleys.

## Materials and Methods

### Plant materials and hydroponic culture

Seeds of cultivated barley cultivars (CM72 and Gairdner) and Tibetan wild barley accessions (XZ16 and XZ169) were disinfected with 3% H_2_O_2_ for 20 min and rinsed with distilled water, then soaked for 12 hours at room temperature. The seeds were transferred onto moist filter papers in the germination boxes in a growth chamber (22/18°C, day/night) in the dark for 3 days, and incubated for another 4 days with light. Seven-days-old seedlings were transplanted into 48-well plastic containers (35 L) with aerated hydroponic solution similar to that used by Wu et al [9]. The pH of the hydroponic solution was adjusted to 6.0 using 1 M HCl, as required. Half concentration solution was supplied to plants in the first week, and then changed into full concentration from the second week. All solutions were renewed weekly. Plants were grown in a greenhouse with natural light, and a temperature of 20±2°C/day and 15±2°C/night at Zijingang Campus, Zhejiang University, China.

### Treatments and sampling

Salt treatment was initiated to plants from the third week by adding NaCl at a rate of 50 mM increase per day, to reach a final concentration of 150 and 300 mM in the solution. The solution without any NaCl addition was used as the control. There were four growth containers as replicates for each treatment (control). Shoots (leaves) were harvested at 35 days after treatment. Tissues of shoots were dried at 80°C for 72 hours, and dry weight was recorded. The relative dry weight was calculated as the ratio of each treated plant to its respective control. Significant difference of data was analyzed by Tukey's studentized range (HSD) test on SAS software (http://www.sas.com/software/sas9/). The statistical difference at P<0.05 is considered as significant. At 21 days after 300 mM salinity, six biological replicates from each treatment were sampled and handled according to the description by Kim and Verpoorte [39], and frozen immediately in liquid nitrogen, and stored at −80°C for use in extraction of metabolites.

### Metabolite extraction and metabolite profiling analysis

Metabolites from barley roots and leaves (100±10 mg of fresh weight) were extracted according to Lisec et al [40] with some modification. Roots and leaves stored at −80°C were ground using liquid nitrogen in a mortar, and transferred into 2 ml centrifuge tubes. 1,400 µl of 100% methanol (pre-cooled at −20°C) was then added and vortexed for 10 s, followed by adding 60 µl of Ribitol (0.2 mg/ml stock in dH_2_O) as an internal quantitative standard and vortexed for 10 s. The tubes were placed into an ultrasound machine at 70°C for 30 min, and then centrifuged for 10 min at 11,000 g and the supernatant was transferred into 5 ml glass centrifuge tubes. After adding 750 µl chloroform (pre-cooled at −20°C) and 1,500 µl deionized water (dH_2_O) (4°C), the tubes were vortexed for 30 s, then centrifuged for 15 min at 2,200 g. 400 µl supernatant was transferred into a new eppendorf tube. Samples were blow-dried by moderate nitrogen. 80 µl of 15 mg^−1^ ml methoxyamine pyridine solution was then added, vortexed for 30 s and reacted for 90 min at 37°C. Finally, 80 µl BSTFA reagent (containing 1% TMCS) was added into the mixture, reacted for 60 min at 70°C. After the above reactions, samples were determined for contents of metabolites using 7890A GC/5975C MS system (Agilent, USA). The programs of temperature-rise was followed by initial temperature of 70°C for 2 min, 10°C/min rate up to 140°C, 4°C/min rate up to 240°C, 10°C/min rate up to 300°C and staying at 300°C for 8 min. Mass spectrometry was determined by full-scan method with range from 50 to 600 (m/z). The raw signals exacting, the data baselines filtering, peak identification and integration were under R software platform (http://cran.r-project.org/). The data were then imported to TagFinder software for correction of retention time to mass debris, peak alignment and deconvolution analysis with default parameters [41]. The total mass of signal integration area was normalized for each sample. That was, the total integral area of each sample was normalized to 1000. Finally, the normalized data were imported into Simca-P software (version 11.5, http://www.umetrics.com/simca), employing PLS-DA model using the first principal component of VIP (variable importance in the projection) values (VIP>1) combined with Student's T test (T-test) (P<0.05) to find differentially expressed metabolites, and search for metabolites from commercial databases such as NIST (http://www.nist.gov/index.html) and KEGG (http://www.genome.jp/kegg/).

### Data analysis and metabolic pathway construction

Before data analysis, all data were standardized using Simca-P software (version 11.5, http://www.umetrics.com/simca). Hierarchical cluster analysis (HCA) and principal component analysis (PCA) models were tested for all samples. Significant differences of metabolites between treatment and control or between genotypes were tested using T-test and ANOVA analysis on SAS software. Quantitative normalization within replicates was transformed by logarithmic base of 2 and Metaboanalyst online analysis software (www.metaboanalyst.ca/) was used to build heatmap diagram [42]. Metabolic pathway was constructed according to pathway analysis on Metaboanalyst, using rice metabolic pathway databases as reference for global test algorithm, according to KEGG metabolic database and constructing metabolic pathways.

## Results

### Growth performance of Tibetan wild barley and cultivated barley under salt stress

**Figure 1:**
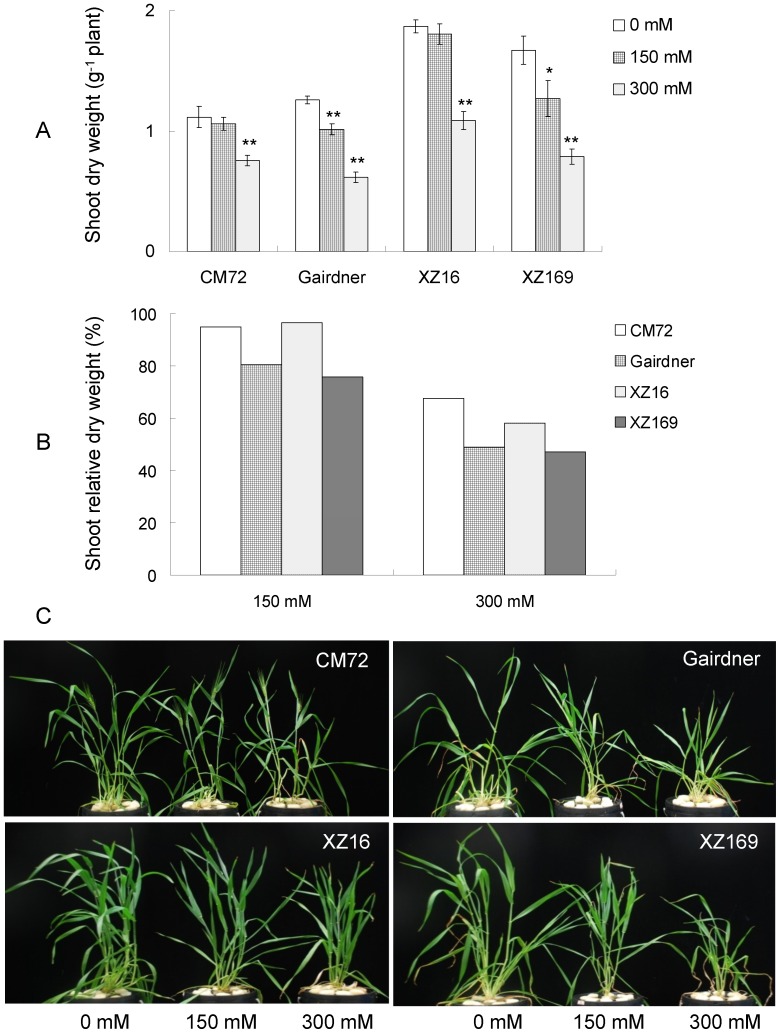
Shoot growth performance of the four barley genotypes (CM72, Gairdner, XZ16 and XZ169) under normal, moderate and high salinity conditions. (A) shoot dry weight (g^−1^ plant) at 35 days after salinity treatments and normal conditions (four biological replicates, bars show SE), * and ** indicates significant (P<0.05) and highly significant difference (P<0.01), respectively; (B) shoot relative dry weight (%) at 35 days after moderate (150 mM NaCl) and high (300 mM) salinity treatment; (C) pictures of shoot-plants of CM72, Gairdner, XZ16 and XZ169 at 35 days after salinity treatment and normal conditions.

**Figure 2:**
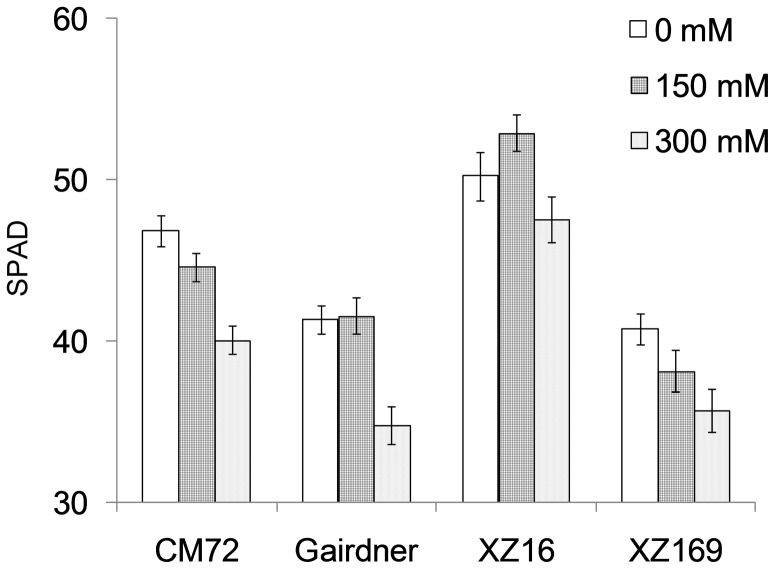
The chlorophyll content (SPAD value) of the four barley genotypes (CM72, Gairdner, XZ16 and XZ169) at 35 days after salinity treatment and normal conditions (eight biological replicates, bars show SE).

In a hydroponic experiment, four barley genotypes exposed to salt stress of 150 and 300 mM NaCl for 35 days, showed distinct difference in salt tolerance as indicated by their biomass at the end of treatment (Fig. 1A). The decrease in shoot dry weight (SDW) caused by moderate concentration (150 mM NaCl) was 12.9%, averaged over the four genotypes, whereas high concentration (300 mM NaCl) caused a reduction of 44.6% (Fig. 1B). Exposed to moderate salinity for 35 days, CM72 and XZ16 did not show significant difference in SDW compared to their controls, while Gairdner and XZ169 deceased by 19.4% and 24.1% in SDW, respectively (Fig. 1B). Under high salinity, CM72 and XZ16 decreased 32.3% and 41.9%, Gairdner and XZ169 decreased by 51.1% and 53%, respectively (Fig. 1B). Among the four tested genotypes, XZ16 maintained the largest absolute shoot biomass under moderate and high salinity conditions, being 1.6 and 1.7 folds larger than the salt tolerant cultivar, CM72 [43], respectively. In addition, the SPAD values of the four genotypes were significantly deceased under high salinity, with XZ16 having the largest SPAD value (Fig. 2), although all genotypes showed symptoms of salt toxicity, characterized by the chlorosis of old leaves and stunted roots, with Gairdner and XZ169 being more affected (Fig. 1C). In short, the current results showed that CM72 and XZ16 were more salt-tolerant than Gairdner and XZ169.

### Metabolic changes in response to salt stress

**Figure 3:**
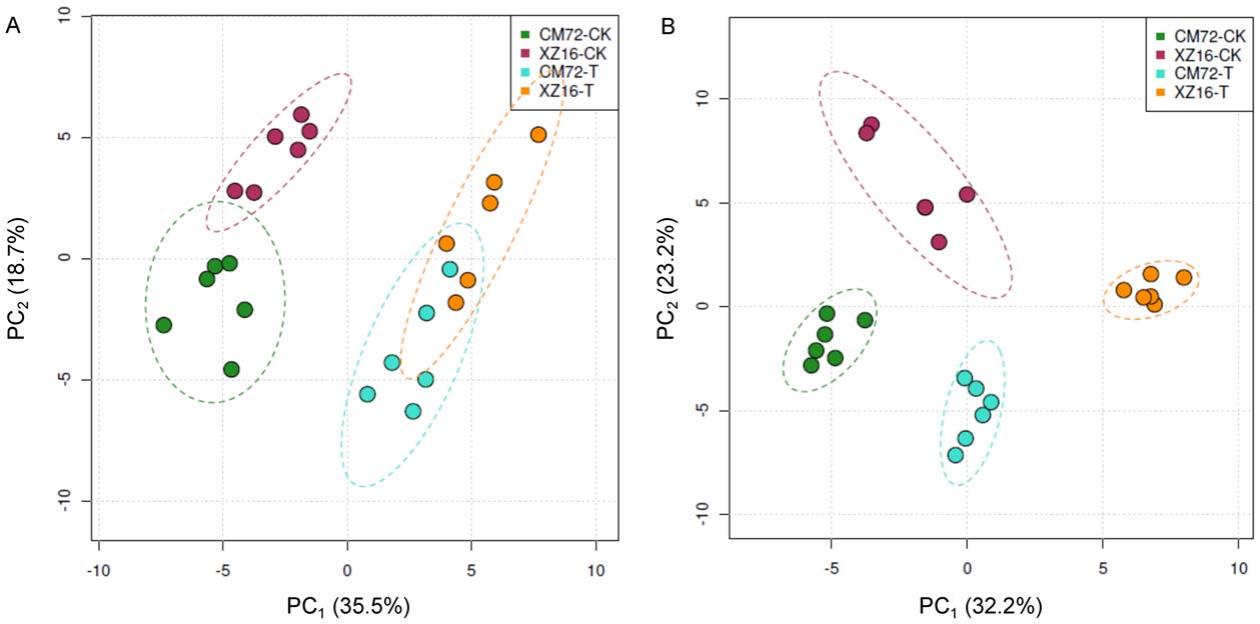
Principal component analysis (PCA) of metabolic profiles in roots and leaves of CM72 and XZ16 under control and high salinity conditions (six biological replicates). (A) PCA in roots; (B) PCA in leaves. CK: control; T: salt treatment; PC1, the first principal component; PC2, the second principal component.

In order to reveal physiological mechanisms of salt tolerance underlying the salt tolerant wild barley, XZ16, metabolic changes in roots and leaves responding to high salinity (300 mM NaCl) were compared with the salt tolerant cultivated barley, CM72. The total ion chromatogram (TIC) of 48 barley samples, including the wild and cultivated barley as well as salt treatment and controls, are shown in File S1. There was an obvious chromatographic difference between sample groups and the retention time was reproducible and stable, indicating the reliability of metabolomic analysis. A total of 82 kinds of metabolites were identified and their concentrations were determined. According to PCA and Heatmap analysis (Fig. 3 and File S1), an obvious separation between samples within treatments and genotypes was detected. The samples of control (i.e. CK) and salt treatment (i.e. T) in both roots and leaves were clearly separated by the PC1, which represented 35.5% and 32.2% of the variation among the samples, respectively. The PC2 distinguished the samples from CM72 and XZ16 in both roots and leaves, explaining 18.7% and 23.2% of the variation, respectively (Fig. 3, A and B). The contribution of metabolites in roots for PC1 came from a number of metabolites dominated by proline, while sucrose and organic acids (e.g. threonine, glyceric acid and isoleucine) was the dominate metabolites contributing to PC2 (File S1). The contribution of metabolites in leaves for PC1 was dominated by inositol and amino acids (e.g. serine, proline and isoleucine), while sugars (e.g. isomaltose, galactose and mannose) and some organic acids were major contributors of PC2 (File S1).

### Difference of metabolic profiles between Tibetan wild barley and cultivated barley under normal conditions

**Figure 4:**
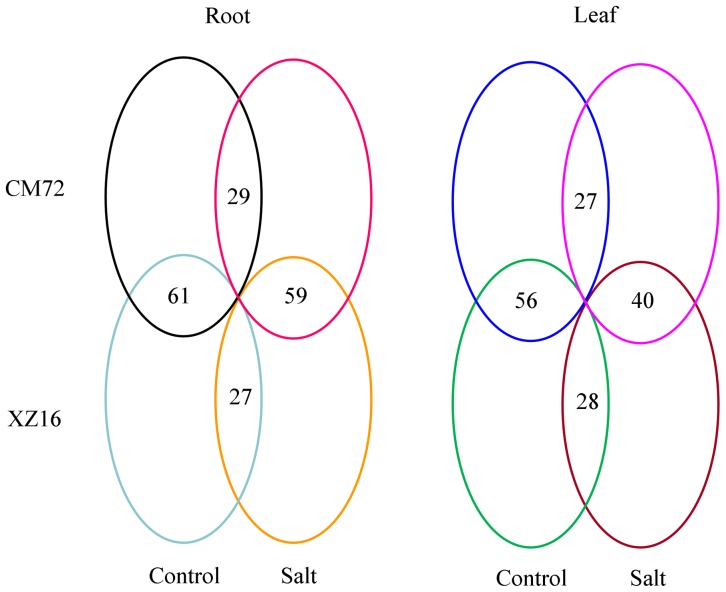
Global comparison of metabolic profiles in roots and leaves between CM72 and XZ16 under control and high salinity conditions. There are eighty-two metabolites identified in this study and the numbers in the figure indicate the numbers of metabolites with no significant difference in their contents for each comparison.

There was a genotypic difference in metabolic profiles under normal condition. The wild barley, XZ16 had significantly (P<0.05) higher contents of 15 metabolites and significantly lower contents of 6 metabolites in roots, comparing with those in roots of CM72 (Fig. 4). These up-accumulated metabolites were mainly amino acids and organic acids (fold was calculated by the formula: log_2_^(XZ16/CM72)^, hereafter the same), including asparagine (1.89-fold), leucine (1.61-fold), isoleucine (1.31-fold), valine (1.03-fold), proline (0.93-fold), threonine (0.77-fold) and serine (0.68-fold); orotic acid (1.40-fold), glyceric acid (0.28-fold) and trans-ferulic acid (0.26-fold) (Table 1 and File S1). Asparagine content showed the largest difference between the two genotypes, indicating the higher capability of wild barley in amino acid biosynthesis and nitrogen storage in roots. In contrast, CM72 was significantly higher in root sugar contents, including affinose (2.39-fold), glucose (1.16-fold) and mannose (0.45-fold) (Table 1 and File S1).

**Table 1:** Relative concentration and fold changes of major metabolites in roots of CM72 and XZ16 after 21 days of 300 mM salinity treatment.

Metabolite name		Relative concentration				Fold changes log_2_^(salt/control)^	
		CM72		XZ16			
		CK	Salt	CK	Salt	CM72	XZ16
Glycolysis	Fructose-6-P	1.57	0.36	1.53	0.48	−2.12**	−1.68**
	Glucose-6-P	4.49	1.11	4.51	1.50	−2.02**	−1.59**
	Glucose	19.26	8.41	8.60	10.83	−1.20**	0.33
	PEP	0.02	0.03	0.02	0.04	0.65	1.23**
	3-PGA	0.22	0.02	0.22	0.08	−3.45**	−1.38*
	Pyruvate	1.30	1.03	1.31	1.16	−0.34	−0.17
Sugars and polyols	Mannose	0.54	0.27	0.39	0.26	−0.98**	−0.60**
	Maltose	0.19	0.21	0.15	0.23	0.10	0.62*
	Raffinose	0.06	0.19	0.01	0.76	1.57	5.95**
	Sucrose	358.30	493.90	333.20	427.00	0.46**	0.36**
	Trehalose	5.06	23.27	5.48	17.14	2.20**	1.64**
	Turanose	0.14	0.29	0.10	0.30	1.10**	1.62**
	Mannitol	0.67	4.78	0.62	3.42	2.84**	2.46**
	Inositol	2.05	3.26	1.89	3.41	0.67**	0.85**
	Xylitol	0.52	2.92	0.43	2.25	2.49**	2.39*
TCA cycle	Citric acid	25.51	61.50	17.23	55.84	1.27**	1.70**
	Fumaric acid	11.98	2.90	7.15	3.92	−2.05**	−0.87
	Isocitric acid	0.24	1.19	0.22	2.23	2.32**	3.34**
	α-Ketoglutaric acid	0.69	0.30	0.77	0.43	−1.18**	−0.86**
	Maleic acid	0.23	0.13	0.21	0.12	−0.86**	−0.80**
	Malic acid	86.73	64.45	79.21	108.40	−0.43	0.45
	Succinic acid	1.25	0.95	1.35	1.34	−0.40	−0.01
Amino acid	Alanine	10.13	4.18	10.52	5.74	−1.28**	−0.88**
	β-alanine	0.92	0.58	1.01	0.74	−0.66**	−0.46**
	Asparagine	5.84	0.28	25.57	0.56	−2.64*	−4.16**
	Aspartate	3.82	1.96	3.27	2.25	−0.96**	−0.54*
	Glutamic acid	9.99	3.54	13.66	8.81	−1.50*	−0.63*
	Glycine	2.96	2.03	2.80	2.58	−0.54**	−0.12
	Isoleucine	1.80	2.47	4.44	3.80	0.46	−0.23
	Leucine	1.07	2.17	3.26	3.23	1.02	−0.01
	Proline	0.12	15.88	0.22	40.31	7.08**	7.50**
	Serine	8.13	11.93	13.05	28.67	0.55	1.13**
	Threonine	2.91	2.49	4.95	5.01	−0.22	0.02
	Valine	10.51	7.29	21.42	7.66	−0.53*	−1.48**

The relative concentration of each metabolite is an average of data from six biological replicates using GC-MS. The fold changes was calculated using the formula log_2_^(salt/control)^.

* and ** indicate significant (P<0.05) and highly significant difference (P<0.01), respectively.

In leaves, there were 16 and 10 metabolites showing significantly higher and lower contents in XZ16 than those in CM72, respectively (Fig. 4). The metabolites with up-accumulation in XZ16 were mainly PEP (4.30-fold), glucose-6-P (1.89-fold), 3-PGA (1.53-fold) and fructose-6-P (1.40-fold), proline (1.43-fold), leucine (1.19-fold) and glutamic acid (0.87-fold) (Table 2 and File S1). While the metabolites showing up-accumulation in CM72 were mainly galactose (1.35-fold), isomaltose (1.24-fold), turanose (0.86-fold), glucose (0.82-fold), mannose (0.71-fold) and maltose (0.49-fold) (Table 2 and File S1).

**Table 2:** Relative concentration and fold changes of major metabolites in leaves of CM72 and XZ16 after 21 days of 300 mM salinity treatment.

Metabolite name		Relative concentration				Fold changes log_2_^(salt/control)^	
		CM72		XZ16			
		CK	Salt	CK	Salt	CM72	XZ16
Glycolysis	Fructose-6-P	0.21	0.53	0.55	0.55	1.35**	0.0
	Glucose-6-P	0.25	0.69	0.92	0.80	1.48**	−0.20
	Glucose	5.78	78.68	3.26	6.55	3.77**	1.00
	PEP	0.00	0.13	0.08	0.10	4.95**	0.36
	3-PGA	0.18	1.62	0.53	1.33	3.15**	1.34**
	Pyruvate	1.49	1.40	1.05	1.30	−0.08	0.30*
Sugars and polyols	Mannose	0.62	4.10	0.38	0.49	2.71**	0.36
	Maltose	0.64	0.37	0.45	0.27	−0.79**	−0.74*
	Raffinose	13.24	43.17	10.23	49.99	1.70**	2.29**
	Sucrose	330.10	229.70	319.90	198.30	−0.52*	−0.69**
	Trehalose	1.81	2.05	1.64	2.34	0.18	0.51
	Turanose	0.33	0.35	0.18	0.20	0.10	0.17
	Mannitol	2.10	0.72	2.15	0.81	−1.54**	−1.42**
	Inositol	15.08	6.92	14.02	4.04	−1.12**	−1.80**
	Xylitol	0.45	0.21	0.61	0.16	−1.11**	−1.93
TCA cycle	Citric acid	33.74	4.57	37.56	5.62	−2.88**	−2.74**
	Fumaric acid	5.06	1.74	7.02	1.31	−1.54**	−2.42**
	Isocitric acid	4.16	6.51	10.03	6.96	0.65	−0.53
	α-Ketoglutaric acid	0.69	0.39	0.81	0.61	−0.82**	−0.41**
	Maleic acid	0.10	0.13	0.11	0.12	0.26	0.07
	Malic acid	83.37	32.09	101.10	22.63	−1.38**	−2.16**
	Succinic acid	1.18	0.48	1.52	0.42	−1.30**	−1.87**
Amino acid	Alanine	17.28	9.75	15.40	17.52	−0.83*	0.19
	β-alanine	0.31	0.32	0.27	0.43	0.05	0.68**
	Asparagine	0.13	2.16	0.99	25.79	1.09*	3.63**
	Aspartate	8.10	3.44	10.43	1.79	−1.24**	−2.54**
	Glutamic acid	22.16	14.52	40.49	23.28	−0.61	−0.80**
	Glycine	8.27	13.13	10.86	19.32	0.67*	0.83**
	Isoleucine	0.21	1.25	0.25	4.50	2.58**	4.17**
	Leucine	0.07	0.58	0.15	4.39	3.13**	4.87**
	Proline	0.01	50.55	0.04	108.30	11.79**	11.47**
	Serine	22.48	73.78	28.16	119.10	1.71**	2.08**
	Threonine	13.65	7.96	10.82	12.55	−0.78**	0.21*
	Valine	3.29	2.52	3.51	10.30	−0.38	1.55**

The relative concentration of each metabolite is an average of data from six biological replicates using GC-MS. The fold changes was calculated using the formula log_2_^(salt/control)^.

* and ** indicate significant (P<0.05) and highly significant difference (P<0.01), respectively.

### Metabolic profiles in response to salt stress in roots

**Figure 5:**
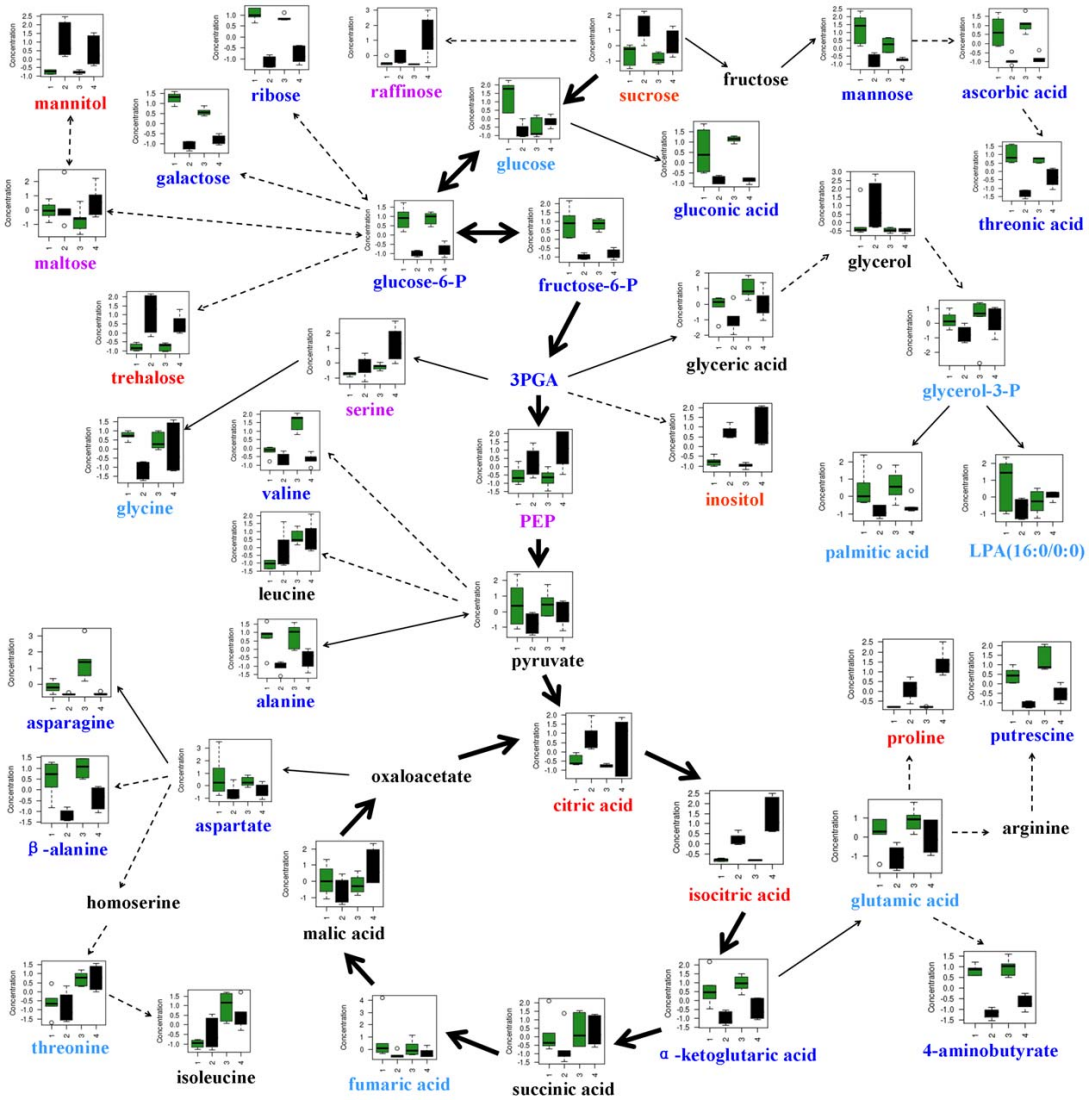
Change in metabolites of the metabolic pathways in roots of CM72 and XZ16 after 21 days of salinity treatment. Numbers 1–4 on the X-axis indicate CM72-control, CM72-salt treatment, XZ16-control and XZ16-salt treatment, respectively. The concentration of metabolites on the Y-axis is presented as normalized values transformed by Metaboanalyst software (www.metaboanalyst.ca/), and the box plots show centered means and standard deviation of each variable. Metabolites in red indicate significant (P<0.05) up-accumulation in both CM72 and XZ16, in purple mean significant (P<0.05) up-accumulation in CM72 or XZ16, in light blue show significant (P<0.05) down-accumulation in CM72 or XZ16, and in dark blue represent significant (P<0.05) down-accumulation in CM72 and XZ16.

According to the results of the PCA (Fig. 3), the response of metabolites to high salinity varied with tissues and genotypes. There were 53 and 55 metabolites with a significant change in root content under salt stress for CM72 and XZ16, respectively (Fig. 4). However some metabolites responded similarly to salt stress in the two genotypes. Under high salinity, accumulation of some kinds of sugar, including sucrose, trehalose and turanose, was enhanced in roots, but fructose-6-P, glucose-6-P and 3-PGA, which are involved in glycolysis, were reduced for both CM72 and XZ16 (Table 1 and Fig. 5). Furthermore, the TCA cycle, associated with glycolysis pathway, was enhanced under salt stress, as shown by higher citric acid and isocitric acid content compared to the control. On the other hand, α-ketoglutaric acid (used for proline synthesis) was depleted dramatically probably due to synthesis of down-stream metabolites (Table 1 and Fig. 5). Aspartate metabolism was inhibited under salt stress, resulting in decrease of asparagine and β-alanine contents. Similarly, pyruvate metabolism was also inhibited, causing a reduction of alanine and valine contents. However, proline, a well-known compatible solute, increased by 7.08 and 7.50 folds (calculated by the formula: log_2_^(salt/control)^) in the roots of CM72 and XZ16 under salt stress, respectively (Table 1 and Fig. 5). In addition, the contents of inositol, mannitol and xylitol was significantly increased, while some organic acids such as ascorbic acid, gluconic acid, orotic acid, pyroglutamic acid and threonic acid were significantly decreased (Table 1 and File S1).

The response of metabolites to salt stress differed between the two barley genotypes. For instance, the contents of PEP, maltose, isomaltose, raffinose and serine were significantly up-accumulated under high salinity only in XZ16 (Table 1 and Fig. 5). Moreover, XZ16 was significantly higher in the contents of 21 metabolites than CM72, including raffinose (1.99-fold), glucose (0.37-fold), proline (1.34-fold), glutamic acid (1.32-fold), serine (1.26-fold), threonine (1.01-fold) and putrescine (1.33-fold). In contrast, two metabolites, with CM72 having higher content, were glycerol (1.78-fold) and ethanolamine (0.49-fold) (File S1). It may be assumed that wild barley has a greater capacity in regulating osmotic stress than cultivated barley by producing more soluble sugars and proline in roots.

### Metabolic profiles in response to salt stress in leaves

**Figure 6:**
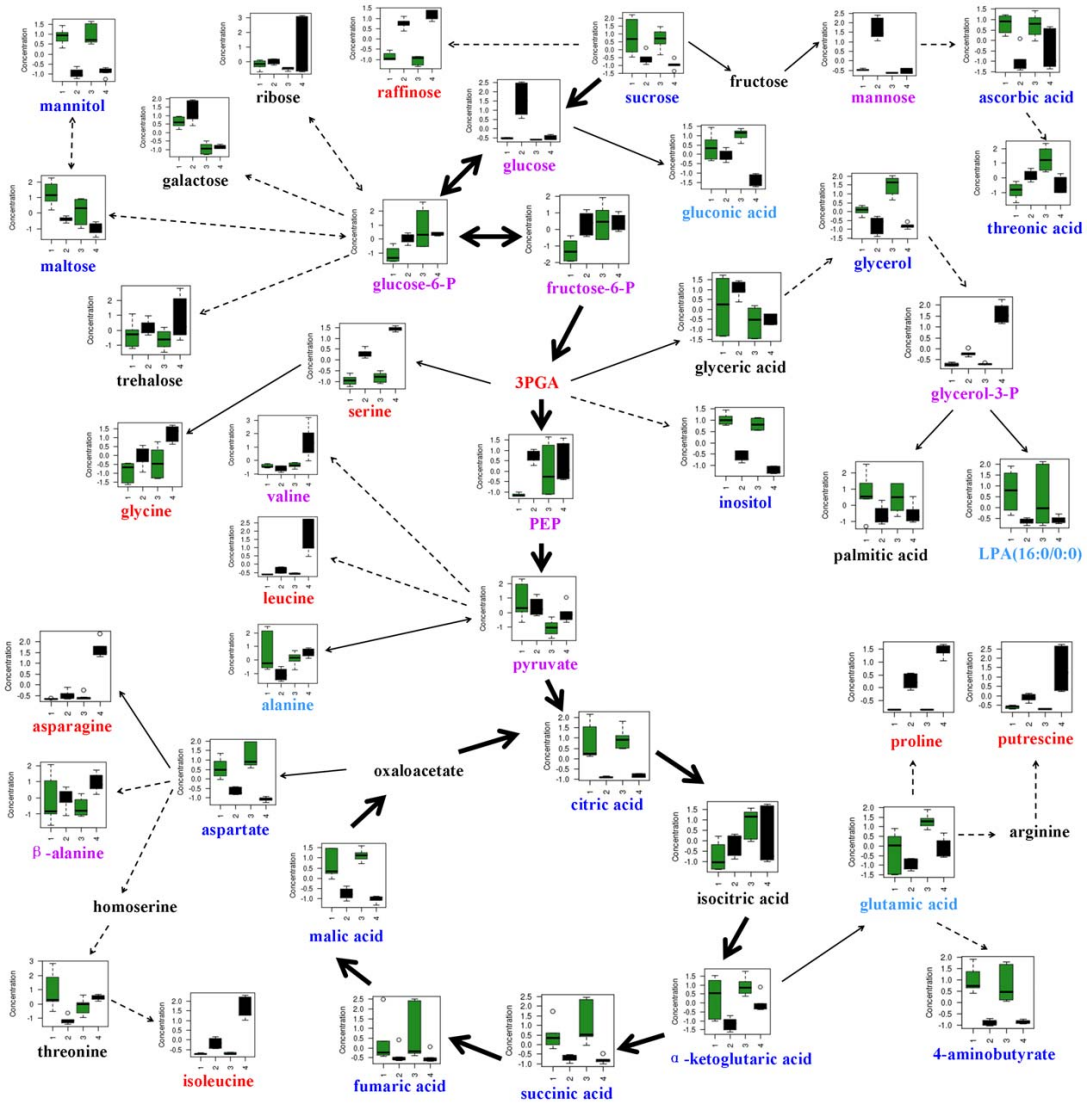
Change in metabolites of the metabolic pathways in leaves of CM72 and XZ16 after 21 days of salinity treatment. Numbers 1–4 on the X-axis indicate CM72-control, CM72-salt treatment, XZ16-control and XZ16-salt treatment, respectively. The concentration of metabolites on the Y-axis is presented as normalized values transformed by Metaboanalyst software (www.metaboanalyst.ca/), and the box plots show centered means and standard deviation of each variable. Metabolites in red indicate significant (P<0.05) up-accumulation in both CM72 and XZ16, in purple mean significant (P<0.05) up-accumulation in CM72 or XZ16, in light blue show significant (P<0.05) down-accumulation in CM72 or XZ16, and in dark blue represent significant (P<0.05) down-accumulation in CM72 and XZ16.

Under salt stress, 23 and 32 metabolites in leaves of CM72, and 20 and 34 metabolites in leaves of XZ16 showed significant up-accumulation and down-accumulation, respectively (Fig. 4). High salinity caused a significant reduction of sucrose content and increased that of some sugars, including isomaltose and raffinose in both CM72 and XZ16. Glycolysis was dramatically enhanced in CM72, but not in XZ16. Hence, glucose, glucose-6-P, fructose-6-P and 3-PGA were significantly up-accumulated in CM72, while pyruvate was significantly up-accumulated only in XZ16. The higher 3-PGA level indicated an enhanced calvin cycle in leaves under high salinity (Table 2 and Fig. 6). In the TCA cycle, citric acid, α-ketoglutaric acid, fumaric acid, malic acid and succinic acid were significantly reduced, indicating that energy production in TCA cycle was affected by high salinity. Meanwhile, asparagine, glycine, isoleucine, leucine, proline and serine were significantly increased (Table 2 and Fig. 6) under salt stress. Proline content was increased by 11.79 and 11.47 folds (calculated by the formula: log_2_^(salt/control)^) in the leaves of CM72 and XZ16, respectively. Furthermore, uracil, diethylphosphate, pipecolic acid and putrescine were also significantly increased, while glucosan, menthol, maltose, mannitol, 4-aminobutyrate, glycerol, inositol, and some organic acids (such as ascorbic acid, benzoic acid, palmitic acid, pyroglutamic acid and threonic acid) were significantly reduced (Table 2 and File S1).

Genotypic difference was also detected in the changes of leaf metabolites under salt stress. In addition to the metabolites involved in glycolysis, which showed obvious up-accumulation in CM72, mannose was also up-accumulated in the cultivated barley (Table 2). XZ16 showed a higher contents of 25 metabolites than CM72, while there were 17 metabolites which were higher in CM72 than in XZ16 (Fig. 4). Most of up-regulated metabolites in XZ16 belonged to amino acids, such as asparagine (3.40-fold), leucine (2.92-fold), valine (2.03-fold), isoleucine (1.85-fold), and proline (1.10-fold), while most of up-accumulated metabolites in CM72 were sugar, including glucose (3.59-fold), mannose (3.07-fold), isomaltose (1.60-fold), galactose (1.53-fold) and arabinose (1.48-fold) (Table 2 and File S1).

## Discussion

The growth of the four barley genotypes (CM72, Gairdner, XZ16 and XZ169) examined in this study was inhibited under salt stress. CM72, recognized as a salt-tolerant cultivar [43], [44] reduced shoot dry weight by 5% and 32.3%, while the salt-tolerant Tibetan wild barley XZ16 reduced shoot dry weight by 4.4% and 41.9% under moderate and high salinity, respectively. Moreover, the absolute dry weight or growth rate should be taken into account when evaluating salt tolerance. XZ16 had greater reduction in terms of relative dry weight than CM72, but it maintained the largest growth rate and absolute dry weight under salt stress. The result suggested that the mechanism of salt tolerance in XZ16 is somewhat different from that of CM72, and the wild barley is attributed to maintenance of higher growth rate under salinity stress. The result was consistent with the findings observed in our pervious study [9].

Osmotic stress affects root functions immediately when plants are subjected to salt stress. Excessive Na accumulation in plant cells causes secondary stress to plants, and salt-tolerant plant, like barley can regulate osmotic stress through many compatible solutes [11], [45]. Proline acts as an osmoprotectant in the plants subjected to osmotic stress, caused by drought or salinity [18], [46]. In this study, proline content increased by more than 2^7^ fold in the roots of CM72 and XZ16, and by more than 2^11^ fold in the leaves of CM72 and XZ16. The similar huge increase of proline contents was reported by Widodo et al. [38] and Chen et al. [47] in the two cultivated barley genotypes. Sugars, such as sucrose, raffinose and trehalose, are also compatible solutes in response to salt stress [16], [17]. Other study has shown sucrose and trehalose increased in response to salt stress in a salt tolerant barley genotype but not in a sensitive one [38]. In this study, sucrose and trehalose in roots, and raffinose in both roots and leaves were significantly up-accumulated in CM72 and XZ16 under salt stress. Mannitol and inositol, well-recognized as osmolytes, also increased under salt stress [21], [48]. Mannitol and inositol, in this study, was up-accumulated in the roots of the plants subjected to salt stress. The rate of mannitol utilization in sink tissues is reported to decrease under salt stress, mainly due to the suppression of the NAD-dependent mannitol dehydrogenase [49], [50]. Accordingly, we suggest that the most important compatible solutes are proline, sugars (sucrose, raffinose and trehalose), mannitol and inositol in roots, and raffinose, proline and some amino acids in leaves.

There was an obvious difference in the metabolites in response to salt stress between tissues as well as genotypes. We found that TCA cycle intermediates and sugar accumulation were enhanced, but glycolysis and amino acid synthesis were inhibited in roots under salt stress. In contrast, calvin cycle, glycolysis and amino acid synthesis appeared to be enhanced in leaves, while TCA cycle was inhibited. The results suggest that high level of sugar content and energy is important for roots to develop salt stress tolerance, and active synthesis metabolism is a basic response for leaves to tolerate salt stress [3], [38], [48]. Osmotic stress takes place in both roots and leaves when plants are exposed to salt stress, while long-term osmotic stress would result in the secondary stress [12], [13]. The difference in the kinds of compatible solutes related to osmotic regulation was found between roots and leaves in the present study. Synthesis of sugars, proline and organic acids was significantly enhanced in roots, being favorable for improvement of the ROS detoxification capacity as well as salt tolerance [45], [47], [51]. However, ascorbic acid recognized as an effective ROS scavenging metabolite was significantly down regulated in roots and leaves of the two genotypes after 21 days of salinity treatment (Fig. 5 and 6). In leaves, a dramatic enhancement of sugars and amino acids synthesis was found, favorable for osmotic adjustment and membrane stability [38], [45], [47]. It could be concluded that proline and raffinose are the common compatible solutes with great changes under salt stress in roots and leaves, while sucrose, trehalose, mannitol and inositol were the root-specific compatible solutes, and some amino acids (asparagine, glycine isoleucine and serine) were leaf-specific solutes.

The changes of metabolites in response to salt stress differed in conservancy and divergence among species or genotypes within the same species [48], [52]. In this study, both CM72 and XZ16 are salt-tolerant. However, the levels of some metabolites differed between the two genotypes under salt stress. Compared with CM72, XZ16 contained lower contents of galactose, glucose and mannose in roots and higher contents of proline, leucine, fructose-6-P, glucose-6-P, PEP and 3-PGA in leaves under normal conditions. Tibetan wild barley, XZ16 exhibited higher capability of amino acid biosynthesis and higher level of glycolysis metabolism, while CM72 showed higher levels of sugar synthesis. Under high salinity, XZ16 had higher proline content in both roots and leaves, and CM72 accumulated more metabolites associated with photosynthesis and TCA cycle in leaves, but less amino acids and organic acids, while the contents of fructose-6-P, glucose-6-P and PEP were not affected by salt stress in leaves of XZ16. It may be suggested that photosynthesis and amino acid synthesis were more affected by salt stress in the cultivated barley than in wild barley [53]. Under salt stress, wild barley had higher contents of glucose, raffinose, glutamic acid and organic acids in roots and higher contents of alanine, asparagine, glycine, isoleucine, serine and raffinose in leaves. These metabolites are commonly considered as compatible solutes, which are involved in osmotic adjustment, protecting membranes and proteins from the damage by ROS [53]–[55].

According to the comparison of metabolic profiles and SPAD value between the two genotypes under control and salt stress, it may be concluded that XZ16 has higher contents of compatible solutes, more active metabolite synthesis and rapid growth than CM72 under salt stress. The wild barleys from the Qinghai-Tibet Plateau, China are a rich source of genetic variation in term of salt stress tolerance [9], [10]. The wild barley accessions, such as XZ16 may have different mechanisms for salinity tolerance from those exists in cultivated barley, thus they are quite useful in salt tolerant improvement of barley and other crops.

## Supporting Information

File S1 **Supplementary Tables and Figures.**
(DOC)
