# Supplementary material for: Tissue Metabolic Responses to Salt Stress in Wild and Cultivated Barley
Source: PLoS One. 2013 Jan 31;8(1):e55431. doi: 10.1371/journal.pone.0055431 (PMC3561194; doi:10.1371/journal.pone.0055431)
Supplement: File S1 — Supplementary Tables and Figures. (DOC) [file pone.0055431.s001.doc]

# Tissue metabolic responses to salt stress in wild and cultivated barley

Dezhi Wu^1^, Shengguan Cai^1^, Mingxian Chen^1^, Lingzhen Ye^1^, Zhonghua Chen^2^, Haitao Zhang^1^, Fei Dai^1^, Feibo Wu**^1^**, Guoping Zhang^1,*^

^1^Department of Agronomy, Key Laboratory of Crop Germplasm Resource of Zhejiang Province, Zhejiang University, Hangzhou 310058, China

^2^School of Science and Health, University of Western Sydney, Penrith 2751 NSW, Australia

^*^Corresponding author: [zhanggp@zju.edu.cn](mailto:zhanggp@zju.edu.cn)

**Table S1. The contribution of metabolites in roots to the first principal component (PC1) and the second principal component (PC2)**

|  | PC1 | PC2 |
| --- | --- | --- |
| 1,6-Anhydroglucose | -0.1899 | 0.0927 |
| LPA(16:0/0:0) | -0.0740 | 0.0126 |
| 2,4,5-Trihydroxy pentanoic acid | -0.0738 | 0.1278 |
| 2,4-dihydroxybutyric acid | 0.1109 | 0.0644 |
| 2,4-dihydroxypyrimidine | -0.1072 | 0.1876 |
| 2-Ketoglutaric acid | -0.1288 | 0.1366 |
| 2-Keto-D-gluconic acid | -0.1746 | 0.1269 |
| (S)-3,4-Dihydroxybutyric acid | 0.0015 | 0.0401 |
| Cinnamic acid | -0.1810 | 0.0707 |
| 4-aminobutyrate | -0.1755 | 0.1400 |
| Trans-Ferulic acid | -0.1748 | 0.1217 |
| 4-hydroxybenzoic acid | -0.0868 | 0.0692 |
| Alanine | -0.1259 | 0.1304 |
| Arabinose | -0.1620 | 0.0557 |
| Asparagine | -0.0933 | 0.1740 |
| Aspartate | -0.1414 | 0.0076 |
| Benzoic acid | -0.1517 | 0.0148 |
| Beta-alanine | -0.1295 | 0.1534 |
| Citric acid | 0.1086 | -0.1066 |
| Menthol | -0.1869 | 0.0091 |
| Quinic acid | -0.1484 | 0.0183 |
| Erythritol | -0.0805 | 0.0894 |
| Ethanolamine | -0.0627 | 0.0353 |
| Fructose-6-phosphate | -0.1833 | 0.1122 |
| Fumaric acid | -0.0962 | 0.0226 |
| Galactinol | 0.1056 | -0.0142 |
| Galactose | -0.1972 | 0.0709 |
| Glucose | -0.1151 | -0.0718 |
| Glucose-6-phosphate | -0.1844 | 0.1205 |
| D-Glutamic acid | -0.0543 | 0.2100 |
| Glyceric acid | -0.0521 | 0.2217 |
| 3-Phosphoglyceric acid | -0.1552 | 0.1158 |
| Glycerol | 0.0293 | -0.1503 |
| Glyceryl monostearate | -0.0698 | -0.0004 |
| Glycine | -0.0947 | 0.1400 |
| Glycolic acid | -0.0137 | 0.0568 |
| Myoinositol | 0.1872 | -0.0527 |
| Isocitric acid | 0.1993 | 0.0186 |
| Isoleucine | 0.0689 | 0.2121 |
| Isomaltose | 0.1594 | 0.0439 |
| L(+)-Ascorbic acid | -0.1777 | 0.1258 |
| L-lactic acid | -0.0488 | -0.0029 |
| Isomaltulose | 0.1612 | -0.0430 |
| Ascorbic acid | -0.1131 | -0.0080 |
| Leucine | 0.1145 | 0.1937 |
| Maleic acid | -0.1887 | 0.0405 |
| Malic acid | 0.0563 | 0.1306 |
| Malonic acid | -0.1215 | 0.0505 |
| Maltose | 0.0736 | -0.0539 |
| Mannitol | 0.1230 | -0.1410 |
| Mannose | -0.1860 | 0.0240 |
| Diethylphosphate | -0.1167 | 0.1792 |
| Methylmalonic acid | -0.1237 | 0.0712 |
| Methylsuccinic acid | -0.1749 | 0.1287 |
| N-Acetyl-L-serine | -0.1181 | 0.1822 |
| Nonanoic acid | -0.1772 | 0.0458 |
| O-Methyl myo inositol | 0.0375 | 0.0005 |
| Orotic acid | -0.1135 | 0.1616 |
| Oxalic acid | -0.1820 | 0.1148 |
| Palmitic acid | -0.1230 | 0.0589 |
| P-coumaric acid | -0.0793 | 0.1482 |
| Phosphoenolpyruvate | 0.1530 | 0.0270 |
| Glycerol 3-phosphate | -0.0080 | 0.1390 |
| Pipecolic acid | 0.1493 | -0.0173 |
| Proline | 0.2014 | 0.0304 |
| 1,3-Diaminopropane | -0.1860 | 0.0052 |
| Putrescine | -0.1412 | 0.1848 |
| Pyroglutamic acid | -0.1444 | 0.1343 |
| Pyruvic acid | -0.1022 | 0.1009 |
| Raffinose | 0.1544 | 0.0460 |
| Ribonic acid | -0.0779 | 0.1851 |
| Ribose | -0.1934 | 0.1099 |
| Serine | 0.1622 | 0.1274 |
| Succinic acid | -0.0229 | 0.1152 |
| Sucrose | 0.0946 | -0.1635 |
| Threonic acid | -0.1570 | 0.0781 |
| Threonine | 0.0572 | 0.2343 |
| Trehalose | 0.1405 | -0.1483 |
| Turanose | 0.1655 | -0.0976 |
| Valine | -0.1046 | 0.1863 |
| Vanillic acid | -0.0925 | 0.1533 |
| Xylitol | 0.1096 | -0.1308 |

**Table S2. The contribution of metabolites in leaves to the first principal component (PC1) and the second principal component** (PC2)

|  | Comp 1 | Comp 2 |
| --- | --- | --- |
| 1,6-Anhydroglucose | -0.1881 | 0.0605 |
| LPA(16:0/0:0) | -0.1354 | 0.0818 |
| 2,4,5-Trihydroxy pentanoic acid | -0.0965 | 0.1456 |
| 2,4-dihydroxybutyric acid | -0.0976 | 0.1866 |
| 2,4-dihydroxypyrimidine | 0.1700 | -0.0711 |
| 2-Ketoglutaric acid | -0.0463 | 0.1515 |
| 2-Keto-D-gluconic acid | -0.1752 | 0.0840 |
| (S)-3,4-Dihydroxybutyric acid | -0.0854 | 0.1457 |
| Cinnamic acid | -0.0252 | -0.0377 |
| 4-aminobutyrate | -0.1541 | 0.0750 |
| Trans-Ferulic acid | -0.0584 | 0.0518 |
| 4-hydroxybenzoic acid | -0.0732 | 0.1433 |
| Alanine | 0.0118 | 0.0753 |
| Arabinose | -0.1015 | -0.1344 |
| Asparagine | 0.2001 | 0.0250 |
| Aspartate | -0.1579 | 0.1092 |
| Benzoic acid | -0.1351 | 0.1397 |
| Beta-alanine | 0.1014 | -0.0056 |
| Citric acid | -0.1456 | 0.1254 |
| Menthol | -0.1548 | 0.1250 |
| Quinic acid | -0.1907 | 0.0901 |
| Erythritol | -0.1245 | 0.0989 |
| Ethanolamine | 0.0601 | 0.0451 |
| Fructose-6-phosphate | 0.1279 | 0.0248 |
| Fumaric acid | -0.0886 | 0.1123 |
| Galactinol | -0.0920 | 0.0227 |
| Galactose | -0.0974 | -0.1880 |
| Glucose | 0.0054 | -0.1651 |
| Glucose-6-phosphate | 0.1093 | 0.0669 |
| D-Glutamic acid | -0.0306 | 0.1866 |
| Glyceric acid | -0.0547 | -0.1187 |
| 3-Phosphoglyceric acid | 0.1380 | -0.1028 |
| Glycerol | -0.1165 | 0.1763 |
| Glyceryl monostearate | -0.1140 | -0.0069 |
| Glycine | 0.1801 | 0.0072 |
| Glycolic acid | 0.0873 | 0.1073 |
| Myoinositol | -0.2008 | 0.0784 |
| Isocitric acid | 0.0336 | 0.0867 |
| Isoleucine | 0.2049 | -0.0074 |
| Isomaltose | 0.0147 | -0.1887 |
| L(+)-Ascorbic acid | -0.1189 | 0.1010 |
| L-lactic acid | -0.0944 | 0.1498 |
| Isomaltulose | 0.1374 | -0.0874 |
| Ascorbic acid | -0.0377 | -0.1631 |
| Leucine | 0.1874 | 0.0166 |
| Maleic acid | 0.0574 | -0.0211 |
| Malic acid | -0.1545 | 0.1330 |
| Malonic acid | 0.0079 | 0.0768 |
| Maltose | -0.1868 | 0.0012 |
| Mannitol | -0.1646 | 0.1352 |
| Mannose | -0.0012 | -0.1718 |
| Diethylphosphate | 0.1930 | -0.0480 |
| Methylmalonic acid | -0.0585 | 0.1642 |
| Methylsuccinic acid | -0.0262 | 0.1806 |
| N-Acetyl-L-serine | -0.0466 | 0.1856 |
| Nonanoic acid | -0.1467 | 0.0821 |
| O-Methyl myo inositol | -0.0696 | 0.1474 |
| Orotic acid | -0.0403 | 0.1480 |
| Oxalic acid | -0.0675 | 0.1595 |
| Palmitic acid | -0.1217 | 0.0707 |
| P-coumaric acid | -0.0570 | 0.1384 |
| Phosphoenolpyruvate | 0.1187 | -0.0420 |
| Glycerol 3-phosphate | 0.2058 | -0.0073 |
| Pipecolic acid | 0.1844 | -0.0751 |
| Proline | 0.2082 | -0.0486 |
| 1,3-Diaminopropane | -0.1551 | 0.1217 |
| Putrescine | 0.1793 | -0.0272 |
| Pyroglutamic acid | -0.0422 | 0.1740 |
| Pyruvic acid | -0.0350 | -0.1444 |
| Raffinose | 0.1798 | -0.1043 |
| Ribonic acid | -0.1850 | 0.0865 |
| Ribose | 0.0736 | -0.0251 |
| Serine | 0.2083 | -0.0538 |
| Succinic acid | -0.1361 | 0.1520 |
| Sucrose | -0.1485 | 0.0557 |
| Threonic acid | -0.1429 | 0.1103 |
| Threonine | -0.0228 | 0.0507 |
| Trehalose | 0.0928 | -0.0405 |
| Turanose | -0.0848 | -0.1691 |
| Valine | 0.1653 | 0.0413 |
| Vanillic acid | -0.0722 | 0.1260 |
| Xylitol | -0.1055 | 0.1270 |

**Table S3. Difference of metabolite profiles in roots and leaves between genotypes under normal and salt conditions**

| Compound name | log_2_^(XZ16/CM72)^ | | | | | | | |
| --- | --- | --- | --- | --- | --- | --- | --- | --- |
|  | leaf-control | | root- control | | leaf-salt | | root-salt | |
| Glucosan | -0.20 | NS | -0.07 | NS | -0.46 | P<0.01 | 0.11 | NS |
| LPA(16:0/0:0) | -0.30 | NS | -0.74 | NS | 0.09 | NS | 0.58 | P<0.05 |
| 2,4,5-Trihydroxy pentanoic acid | 0.40 | NS | 0.15 | NS | -0.10 | NS | 0.08 | NS |
| 2,4-dihydroxybutyric acid | 0.71 | P<0.05 | -0.66 | P<0.05 | 0.44 | P<0.05 | 1.11 | NS |
| Uracil | 1.07 | P<0.05 | 0.88 | P<0.05 | -0.05 | NS | 0.38 | NS |
| α-Ketoglutaric acid | 0.23 | NS | 0.17 | NS | 0.64 | P<0.01 | 0.50 | NS |
| Gluconic acid | 0.28 | P<0.05 | 0.32 | NS | -0.92 | P<0.01 | -0.04 | NS |
| 3,4-Dihydroxybutyric acid | 1.19 | NS | 0.42 | NS | -0.46 | NS | 0.06 | NS |
| Sinapate | -0.59 | P<0.05 | -0.20 | NS | 0.25 | P<0.05 | -0.08 | NS |
| 4-aminobutyrate | -0.13 | NS | 0.07 | NS | 0.08 | NS | 0.59 | P<0.05 |
| Trans-Ferulic acid | 0.14 | NS | 0.26 | P<0.05 | -0.14 | NS | -0.16 | NS |
| 4-hydroxybenzoic acid | 1.27 | NS | 0.27 | NS | -0.41 | NS | -0.10 | NS |
| Alanine | -0.17 | NS | 0.05 | NS | 0.85 | P<0.01 | 0.46 | NS |
| Arabinose | -0.29 | NS | 0.06 | NS | -1.48 | P<0.01 | -0.16 | NS |
| Asparagine | 0.86 | NS | 1.89 | P<0.01 | 3.40 | P<0.01 | 0.37 | NS |
| Aspartate | 0.37 | NS | -0.22 | NS | -0.94 | P<0.01 | 0.20 | NS |
| Benzoic acid | 0.23 | NS | -0.10 | NS | -0.08 | NS | -0.05 | NS |
| β-alanine | -0.21 | NS | 0.14 | NS | 0.43 | P<0.05 | 0.34 | P<0.01 |
| Citric acid | 0.16 | NS | -0.57 | P<0.05 | 0.30 | NS | -0.14 | NS |
| Menthol | 0.16 | NS | -0.09 | NS | -0.15 | NS | -0.24 | NS |
| Quinic acid | 0.22 | NS | -0.36 | NS | -2.23 | P<0.01 | 0.25 | P<0.01 |
| Erythritol | -0.22 | NS | 0.00 | NS | -0.04 | NS | 0.24 | NS |
| Ethanolamine | -0.03 | NS | 0.38 | P<0.01 | 0.14 | NS | -0.49 | P<0.05 |
| Fructose-6-phosphate | 1.40 | P<0.01 | -0.04 | NS | 0.05 | NS | 0.40 | NS |
| Fumaric acid | 0.47 | NS | -0.74 | NS | -0.41 | NS | 0.43 | NS |
| Galactinol | -0.43 | NS | -0.21 | NS | 0.24 | P<0.01 | 0.19 | NS |
| Galactose | -1.35 | P<0.01 | -0.37 | P<0.01 | -1.53 | P<0.01 | 0.41 | NS |
| Glucose | -0.82 | P<0.01 | -1.16 | P<0.01 | -3.59 | P<0.01 | 0.37 | P<0.05 |
| Glucose-6-phosphate | 1.89 | P<0.01 | 0.01 | NS | 0.21 | NS | 0.44 | NS |
| Glutamic acid | 0.87 | P<0.01 | 0.45 | NS | 0.68 | P<0.05 | 1.32 | P<0.05 |
| Glyceric acid | -0.42 | NS | 0.28 | P<0.05 | -0.70 | P<0.01 | 0.30 | NS |
| 3-Phosphoglyceric acid | 1.53 | P<0.01 | -0.05 | NS | -0.28 | NS | 2.02 | NS |
| Glycerol | 0.62 | P<0.01 | -0.76 | NS | -0.05 | NS | -1.78 | P<0.05 |
| Glyceryl monostearate | -1.54 | NS | -1.50 | NS | -0.40 | NS | 1.19 | NS |
| Glycine | 0.39 | NS | -0.08 | NS | 0.56 | P<0.01 | 0.34 | NS |
| Glycolic acid | 0.72 | NS | 0.04 | NS | 0.45 | P<0.01 | 0.18 | NS |
| inositol | -0.10 | NS | -0.11 | NS | -0.78 | P<0.01 | 0.07 | NS |
| Isocitric acid | 1.27 | P<0.01 | -0.11 | NS | 0.10 | NS | 0.91 | P<0.05 |
| Isoleucine | 0.26 | NS | 1.31 | P<0.01 | 1.85 | P<0.01 | 0.62 | P<0.05 |
| Isomaltose | -1.24 | P<0.01 | -0.02 | NS | -1.60 | P<0.01 | 0.79 | P<0.05 |
| Ascorbic acid | -0.03 | NS | 0.26 | NS | 0.49 | NS | 0.36 | NS |
| Lactic acid | 0.71 | P<0.05 | 0.14 | NS | -0.02 | NS | -0.05 | NS |
| Isomaltulose | -0.27 | NS | -0.21 | P<0.05 | 0.13 | NS | 0.30 | NS |
| Leucine | 1.19 | P<0.01 | 1.61 | P<0.01 | 2.92 | P<0.01 | 0.58 | NS |
| Maleic acid | 0.13 | NS | -0.15 | NS | -0.07 | NS | -0.10 | NS |
| Malic acid | 0.28 | NS | -0.13 | NS | -0.50 | NS | 0.75 | P<0.05 |
| Malonic acid | 0.20 | NS | -0.23 | NS | 0.33 | P<0.05 | 0.30 | P<0.05 |
| Maltose | -0.49 | P<0.05 | -0.35 | NS | -0.45 | P<0.01 | 0.17 | NS |
| Mannitol | 0.03 | NS | -0.10 | NS | 0.16 | NS | -0.48 | NS |
| Mannose | -0.71 | P<0.01 | -0.45 | P<0.05 | -3.07 | P<0.01 | -0.07 | NS |
| Diethylphosphate | 0.49 | NS | 0.92 | P<0.05 | 0.64 | P<0.05 | 0.71 | P<0.01 |
| Methylmalonic acid | 0.81 | NS | 0.13 | NS | 0.52 | NS | -0.12 | NS |
| Methylsuccinic acid | 0.31 | NS | 0.17 | NS | 0.58 | P<0.01 | 0.24 | P<0.05 |
| N-Acetylserine | 0.26 | NS | 0.69 | P<0.05 | 0.34 | P<0.05 | 0.34 | NS |
| pelargonic acid | -0.05 | NS | 0.01 | NS | -0.13 | NS | -0.08 | NS |
| Sequoyitol | 1.69 | NS | 0.50 | NS | -0.10 | NS | 0.05 | NS |
| Orotic acid | 3.47 | P<0.01 | 1.40 | P<0.01 | 0.26 | NS | -0.27 | NS |
| Oxalic acid | 0.80 | P<0.01 | 0.09 | NS | 0.41 | P<0.01 | 0.10 | NS |
| Palmitic acid | -0.11 | NS | 0.11 | NS | 0.08 | NS | -0.04 | NS |
| 4-hydroxycinnamic acid | 1.19 | NS | -0.08 | NS | -0.52 | P<0.01 | 0.76 | P<0.01 |
| Phosphoenolpyruvate | 4.30 | P<0.05 | -0.14 | NS | -0.29 | NS | 0.44 | NS |
| Glycerol 3-phosphate | 0.11 | P<0.05 | 0.04 | NS | 1.79 | P<0.01 | 0.50 | NS |
| Pipecolic acid | -0.04 | NS | -0.12 | NS | 0.41 | NS | 0.06 | NS |
| Proline | 1.43 | P<0.05 | 0.93 | P<0.01 | 1.10 | P<0.01 | 1.34 | P<0.01 |
| 1,3-Diaminopropane | 0.11 | NS | -0.13 | NS | -0.10 | NS | -0.22 | NS |
| Putrescine | -0.86 | P<0.05 | 0.48 | P<0.05 | 1.63 | P<0.01 | 1.33 | P<0.01 |
| Pyroglutamic acid | 0.35 | NS | 0.26 | NS | 0.69 | P<0.01 | 0.70 | P<0.01 |
| Pyruvic acid | -0.50 | P<0.01 | 0.01 | NS | -0.12 | NS | 0.17 | NS |
| Raffinose | -0.37 | NS | -2.39 | NS | 0.21 | P<0.05 | 1.99 | P<0.05 |
| Ribonic acid | 0.14 | NS | 0.40 | NS | -0.67 | P<0.01 | 0.39 | P<0.05 |
| Ribose | -0.21 | NS | -0.07 | NS | 0.39 | NS | 0.24 | NS |
| Serine | 0.32 | NS | 0.68 | P<0.01 | 0.69 | P<0.01 | 1.26 | P<0.05 |
| Succinic acid | 0.37 | NS | 0.12 | NS | -0.19 | NS | 0.50 | NS |
| Sucrose | -0.05 | NS | -0.10 | NS | -0.21 | P<0.05 | -0.21 | NS |
| Threonic acid | 0.22 | P<0.01 | -0.17 | NS | -0.39 | P<0.05 | -0.05 | NS |
| Threonine | -0.33 | NS | 0.77 | P<0.01 | 0.66 | P<0.01 | 1.01 | P<0.01 |
| Trehalose | -0.14 | NS | 0.12 | NS | 0.19 | NS | -0.44 | NS |
| Turanose | -0.86 | P<0.01 | -0.46 | NS | -0.78 | P<0.01 | 0.05 | NS |
| Valine | 0.09 | NS | 1.03 | P<0.01 | 2.03 | P<0.01 | 0.07 | NS |
| Vanillic acid | 0.49 | NS | 0.10 | NS | -0.16 | NS | 0.34 | NS |
| Xylitol | 0.44 | NS | -0.28 | NS | -0.38 | NS | -0.38 | NS |

The data was calculated using the formula log_2_^(XZ16/CM72)^. P<0.05 and P<0.01 mean significant and highly significant difference, respectively; NS means non-significant difference.

**Table S4. Metabolite profiles changes in roots and leaves of CM72 and XZ16 after 21 days salt treatment**

| Compound name | log_2_^(salt/control)^ | | | | | | | |
| --- | --- | --- | --- | --- | --- | --- | --- | --- |
|  | CM72  leaf | | XZ16  leaf | | CM72  root | | XZ16  root | |
| Glucosan | -0.58 | P<0.01 | -0.85 | P<0.01 | -1.10 | P<0.01 | -0.91 | P<0.01 |
| LPA(16:0/0:0) | -1.98 | P<0.01 | -1.59 | NS | -1.04 | P<0.05 | 0.27 | NS |
| 2,4,5-Trihydroxy pentanoic acid | -0.10 | NS | -0.60 | P<0.01 | -0.24 | NS | -0.31 | P<0.05 |
| 2,4-dihydroxybutyric acid | -1.01 | P<0.01 | -1.28 | P<0.01 | -0.23 | NS | 1.54 | P<0.05 |
| Uracil | 2.44 | P<0.01 | 1.31 | P<0.01 | -1.04 | P<0.05 | -1.54 | P<0.01 |
| α-Ketoglutaric acid | -0.82 | P<0.01 | -0.41 | P<0.01 | -1.18 | P<0.01 | -0.86 | P<0.01 |
| Gluconic acid | -0.22 | NS | -1.41 | P<0.01 | -1.22 | P<0.01 | -1.58 | P<0.01 |
| 3,4-Dihydroxybutyric acid | -1.03 | NS | -2.68 | NS | 0.19 | NS | -0.17 | NS |
| Sinapate | -0.46 | NS | 0.38 | P<0.01 | -0.77 | P<0.01 | -0.65 | NS |
| 4-aminobutyrate | -2.13 | P<0.01 | -1.93 | P<0.01 | -1.63 | P<0.01 | -1.11 | P<0.01 |
| Trans-Ferulic acid | 0.05 | NS | -0.24 | NS | -0.66 | P<0.01 | -1.08 | P<0.01 |
| 4-hydroxybenzoic acid | -0.22 | NS | -1.91 | NS | -0.20 | NS | -0.57 | NS |
| Alanine | -0.83 | P<0.05 | 0.19 | NS | -1.28 | P<0.01 | -0.88 | P<0.01 |
| Arabinose | 0.54 | P<0.05 | -0.65 | P<0.01 | -0.49 | P<0.01 | -0.71 | P<0.05 |
| Asparagine | 1.09 | P<0.05 | 3.63 | P<0.01 | -2.64 | P<0.05 | -4.16 | P<0.01 |
| Aspartate | -1.24 | P<0.01 | -2.54 | P<0.01 | -0.96 | P<0.01 | -0.54 | P<0.05 |
| Benzoic acid | -0.66 | P<0.01 | -0.96 | P<0.05 | -0.44 | P<0.05 | -0.39 | NS |
| β-alanine | 0.05 | NS | 0.68 | P<0.01 | -0.66 | P<0.01 | -0.46 | P<0.01 |
| Citric acid | -2.88 | P<0.01 | -2.74 | P<0.01 | 1.27 | P<0.01 | 1.70 | NS |
| Menthol | -0.61 | P<0.01 | -0.92 | P<0.01 | -0.50 | P<0.05 | -0.65 | P<0.01 |
| Quinic acid | -0.87 | P<0.01 | -3.32 | P<0.01 | -1.03 | P<0.05 | -0.42 | NS |
| Erythritol | -1.42 | P<0.05 | -1.25 | P<0.05 | -0.66 | NS | -0.42 | NS |
| Ethanolamine | -0.07 | NS | 0.10 | NS | 0.23 | NS | -0.64 | P<0.01 |
| Fructose-6-phosphate | 1.35 | P<0.01 | 0 | NS | -2.12 | P<0.01 | -1.68 | P<0.01 |
| Fumaric acid | -1.54 | NS | -2.42 | NS | -2.05 | NS | -0.87 | NS |
| Galactinol | -0.66 | P<0.05 | 0.01 | NS | 0.23 | NS | 0.63 | P<0.05 |
| Galactose | 0.28 | NS | 0.10 | NS | -1.98 | P<0.01 | -1.21 | P<0.01 |
| Glucose | 3.77 | P<0.01 | 1 | NS | -1.20 | P<0.01 | 0.33 | NS |
| Glucose-6-phosphate | 1.48 | P<0.01 | -0.20 | NS | -2.02 | P<0.01 | -1.59 | P<0.01 |
| Glutamic acid | -0.61 | NS | -0.8 | P<0.01 | -1.50 | P<0.05 | -0.63 | P<0.05 |
| Glyceric acid | 0.33 | NS | 0.05 | NS | -0.26 | NS | -0.23 | NS |
| 3-Phosphoglyceric acid | 3.15 | P<0.01 | 1.34 | P<0.05 | -3.45 | P<0.01 | -1.38 | P<0.05 |
| Glycerol | -0.52 | P<0.01 | -1.19 | P<0.01 | 0.99 | NS | -0.03 | NS |
| Glyceryl monostearate | -2.64 | NS | -1.50 | P<0.05 | -2.18 | NS | 0.51 | NS |
| Glycine | 0.67 | P<0.05 | 0.83 | P<0.01 | -0.54 | P<0.01 | -0.12 | NS |
| Glycolic acid | 0.48 | P<0.05 | 0.21 | NS | -0.09 | NS | 0.04 | NS |
| inositol | -1.12 | P<0.01 | -1.80 | P<0.01 | 0.67 | P<0.01 | 0.85 | P<0.01 |
| Isocitric acid | 0.65 | NS | -0.53 | NS | 2.32 | P<0.01 | 3.34 | P<0.01 |
| Isoleucine | 2.58 | P<0.01 | 4.17 | P<0.01 | 0.46 | NS | -0.23 | NS |
| Isomaltose | 1.63 | P<0.01 | 1.27 | P<0.01 | 0.41 | NS | 1.22 | P<0.01 |
| Ascorbic acid | -1.44 | P<0.01 | -0.92 | P<0.05 | -2.25 | P<0.01 | -2.14 | P<0.01 |
| Lactic acid | -0.70 | P<0.05 | -1.43 | NS | 0 | NS | -0.19 | NS |
| Isomaltulose | 0.65 | P<0.01 | 1.05 | P<0.01 | 0.47 | P<0.05 | 0.98 | P<0.01 |
| Leucine | 3.13 | P<0.01 | 4.87 | P<0.01 | 1.02 | NS | -0.01 | NS |
| Maleic acid | 0.26 | NS | 0.07 | NS | -0.86 | P<0.01 | -0.80 | P<0.01 |
| Malic acid | -1.38 | P<0.01 | -2.16 | P<0.01 | -0.43 | NS | 0.45 | NS |
| Malonic acid | -0.28 | NS | -0.16 | NS | -0.84 | P<0.01 | -0.32 | NS |
| Maltose | -0.79 | P<0.01 | -0.74 | P<0.05 | 0.10 | NS | 0.62 | P<0.05 |
| Mannitol | -1.54 | P<0.01 | -1.42 | P<0.01 | 2.84 | P<0.01 | 2.46 | P<0.01 |
| Mannose | 2.71 | P<0.01 | 0.36 | NS | -0.98 | P<0.01 | -0.60 | P<0.01 |
| Diethylphosphate | 1.28 | P<0.01 | 1.43 | P<0.01 | -1.53 | P<0.05 | -1.74 | P<0.01 |
| Methylmalonic acid | -0.71 | NS | -1 | NS | -0.44 | NS | -0.68 | P<0.05 |
| Methylsuccinic acid | -0.52 | P<0.01 | -0.25 | NS | -0.84 | P<0.01 | -0.77 | P<0.01 |
| N-Acetylserine | -0.41 | P<0.05 | -0.32 | P<0.05 | -1.05 | P<0.01 | -1.40 | P<0.01 |
| pelargonic acid | -0.56 | P<0.05 | -0.64 | P<0.01 | -0.43 | P<0.05 | -0.52 | P<0.01 |
| Sequoyitol | -0.85 | NS | -2.64 | NS | 0.71 | NS | 0.26 | NS |
| Orotic acid | -0.67 | NS | -3.88 | P<0.01 | -1.49 | P<0.01 | -3.15 | P<0.01 |
| Oxalic acid | -0.83 | P<0.01 | -1.22 | P<0.01 | -0.90 | P<0.01 | -0.89 | P<0.01 |
| Palmitic acid | -0.77 | P<0.05 | -0.59 | P<0.05 | -0.70 | NS | -0.85 | P<0.05 |
| 4-hydroxycinnamic acid | 0.55 | P<0.01 | -1.15 | P<0.05 | -0.99 | P<0.01 | -0.15 | NS |
| Phosphoenolpyruvate | 4.95 | P<0.01 | 0.36 | NS | 0.65 | NS | 1.23 | P<0.01 |
| Glycerol 3-phosphate | 1.69 | P<0.01 | 3.37 | P<0.01 | -0.51 | P<0.05 | -0.04 | NS |
| Pipecolic acid | 1.94 | P<0.01 | 2.38 | P<0.01 | 1.16 | P<0.05 | 1.34 | P<0.01 |
| Proline | 11.79 | P<0.01 | 11.47 | P<0.01 | 7.08 | P<0.01 | 7.50 | P<0.01 |
| 1,3-Diaminopropane | -0.66 | P<0.01 | -0.86 | P<0.01 | -0.54 | P<0.05 | -0.63 | P<0.01 |
| Putrescine | 1.65 | P<0.01 | 4.15 | P<0.01 | -2.28 | P<0.01 | -1.43 | P<0.01 |
| Pyroglutamic acid | -0.79 | P<0.01 | -0.45 | P<0.01 | -1.75 | P<0.01 | -1.31 | P<0.01 |
| Pyruvic acid | -0.08 | NS | 0.30 | P<0.05 | -0.34 | NS | -0.17 | NS |
| Raffinose | 1.70 | P<0.01 | 2.29 | P<0.01 | 1.57 | NS | 5.95 | P<0.01 |
| Ribonic acid | -0.43 | P<0.05 | -1.25 | P<0.01 | -0.47 | NS | -0.49 | P<0.01 |
| Ribose | 0.11 | NS | 0.71 | NS | -1.35 | P<0.01 | -1.04 | P<0.01 |
| Serine | 1.71 | P<0.01 | 2.08 | P<0.01 | 0.55 | NS | 1.13 | P<0.01 |
| Succinic acid | -1.3 | P<0.01 | -1.87 | P<0.01 | -0.40 | NS | -0.01 | NS |
| Sucrose | -0.52 | P<0.05 | -0.69 | P<0.01 | 0.46 | P<0.01 | 0.36 | P<0.01 |
| Threonic acid | -0.24 | P<0.01 | -0.84 | P<0.01 | -1.33 | P<0.01 | -1.20 | P<0.01 |
| Threonine | -0.78 | P<0.01 | 0.21 | P<0.05 | -0.22 | NS | 0.02 | NS |
| Trehalose | 0.18 | NS | 0.51 | NS | 2.20 | P<0.01 | 1.64 | P<0.01 |
| Turanose | 0.10 | NS | 0.17 | NS | 1.10 | P<0.01 | 1.62 | P<0.01 |
| Valine | -0.38 | NS | 1.55 | P<0.01 | -0.53 | P<0.05 | -1.48 | P<0.01 |
| Vanillic acid | -0.17 | NS | -0.82 | NS | -0.67 | P<0.05 | -0.43 | P<0.05 |
| Xylitol | -1.11 | P<0.01 | -1.93 | NS | 2.49 | P<0.01 | 2.39 | P<0.05 |

The data was calculated using the formula log_2_^(salt/control)^. P<0.05 and P<0.01 mean significant and highly significant difference, respectively; NS means non-significant difference.

**Figure S1. Total ion chromatogram (TIC) of the forty-eight samples of barley scanned based on GC-MS**

1: CM72 (leaf, control); 2. CM72 (root, control); 3: XZ16 (leaf, control); 4. XZ16 (root, control); 5: CM72 (leaf, salt); 6. CM72 (root, salt); 7: XZ16 (leaf, salt); 8. XZ16 (root, salt); and 6 replicates for each group.

**Figure S2. Heatmap analysis combined with hierarchical cluster analysis (HCA) of eighty-two metabolites**

(A) CM72 (leaf, control); (B) XZ16 (leaf, control); (C) CM72 (root, control); (D) XZ16 (root, control); (E) CM72 (leaf, salt); (F) XZ16 (leaf, salt); (G) CM72 (root, salt); and (H) XZ16 (root, salt). Samples are color coded as the legend on the figure.

1-10：3-Phosphoglyceric acid (3-PGA)，Phosphoenolpyruvate (PEP)，Mannose，Glucose，Isomaltose，Isomaltulose，Serine，Raffinose，Glycine，Glycerol-3-Phosphate；11-20：Proline，Glutamic acid，L-Ascorbic acid，Isocitric acid，Orotic acid，Galactinol，Threonine，Alanine，Threonic acid，Aspartate；21-30：Ribonic acid，Turanose，Inositol，Maltose，Galactose，Ascorbic acid，Glyceric acid，Pipecolic acid，Sucrose，Trehalose；31-40：Mannitol，Xylitol，Citric acid，Glycerol，2,4-dihydroxybutyric acid，Valine，Diethylphosphate，Putrescine，Asparagine，Leucine；41-50：Isoleucine，lactic acid，4-hydroxybenzoic acid，Methyl inositol，3,4-Dihydroxybutyric acid，Glycolic acid，Maleic acid，Gluconic acid, Fructose-6-Phosphate (Fructose-6-P)，Glucose-6-Phosphate (Glucose-6-P)；51-60：Arabinose，β-alanine，Ribose，Ethanolamine，4-hydroxycinnamic acid，2,4,5-Trihydroxy pentanoic acid，Quinic acid，Oxalic acid，4-aminobutyrate (GABA)，glucosan；61-70：Methylsuccinic acid，Trans-Ferulic acid，Palmitic acid，Benzoic acid，Menthol，1,3-Diaminopropane，Nonanoic acid，Succinic acid，Fumaric acid，LPA(16:0/0:0)；71-80：Methylmalonic acid，Vanillic acid，Erythritol，Malic acid，Uracil，α-Ketoglutaric acid，Pyroglutamic acid，N-Acetylserine，Malonic acid，Glyceryl monostearate；81：Sinapate；82：Pyruvate.
